# Ameliorative effects and mechanism of *Panax notoginseng* extract on ulcerative colitis mice based on a multi-omics strategy

**DOI:** 10.3389/fimmu.2025.1727993

**Published:** 2025-12-15

**Authors:** Yan Song, Rui Lin, Yifeng Fu, Anna Wang, Hu Yang, Wentian Liu

**Affiliations:** 1 Department of Gastroenterology and Hepatology, Tianjin Medical University General Hospital, Tianjin, China; 2 College of Bioscience and Biotechnology, Hunan Agricultural University, Changsha, Hunan, China; 3 Department of Nephrology, Tianjin Medical University No.2 Hospital, Tianjin, China

**Keywords:** ulcerative colitis, gut microbes, *P. notoginseng* extract, intestinal barrier, health effects, natural compounds

## Abstract

**Introduction:**

As a complex and persistent inflammatory bowel disease, the onset and progression of ulcerative colitis (UC) are closely associated with intestinal microbiota dysbiosis, host metabolic imbalance, and impaired intestinal barrier function. The traditional Chinese medicine *Panax notoginseng* (Sanqi) possesses multiple therapeutic properties, among which its anti-inflammatory effect is particularly remarkable. However, the specific molecular pathways through which *Panax notoginseng* exerts its anti-UC effects have not been fully elucidated. This study aims to clarify the efficacy and molecular mechanisms of *Panax notoginseng* extract in a mouse model of UC.

**Methods:**

A colitis model was established by inducing UC in ICR mice using dextran sulfate sodium (DSS). The experimental animals were divided into four groups: normal control group (CON), normal administration group (CONSQ), DSS-induced model group (DSS), and DSS-induced administration group (DSSSQ). The CONSQ and DSSSQ groups received oral gavage of 200 mg/kg *Panax notoginseng* extract. The evaluation indicators included the disease activity index, histopathological examination of colon tissue, expression of key intestinal barrier proteins, analysis of intestinal microbiota structure, and metabolomic testing of fecal samples.

**Results:**

Treatment with *Panax notoginseng* extract repaired the damaged intestinal barrier, as evidenced by increased expression levels of Claudin-1, Occludin, ZO-1, and MUC-2 proteins. Simultaneously, the extract favorably modulated the structure of the intestinal microbiota, specifically by increasing the Firmicutes/Bacteroidetes ratio and enriching probiotic genera (such as Bifidobacterium and Lactobacillus). Furthermore, the extract significantly reduced the levels of characteristic metabolites (such as LysoPI and Etamiphylline). Correlation analysis based on multi-omics data revealed an interactive regulatory network centered on the intestinal microbiota, host metabolites, and intestinal barrier integrity, indicating that *Panax notoginseng* extract alleviates the pathological process of UC through a multi-target, synergistic approach.

**Discussion:**

The results of this study demonstrate that *Panax notoginseng* extract exerts its therapeutic effects on UC by repairing the intestinal barrier, modulating the composition of the intestinal microbiota, and influencing the host metabolic profile. Multi-omics correlation analysis further revealed the central role of the microbiota–metabolite–barrier axis in the anti-UC effects of *Panax notoginseng*, providing strong evidence for its multi-target synergistic mechanism. These findings lay the foundation for a deeper understanding of the pharmacological mechanisms of Panax notoginseng in UC treatment and support its further development as a potential therapeutic agent for UC.

## 1 Introduction

The global incidence of ulcerative colitis (UC), a chronic inflammatory disorder of the bowel, is increasingly prevalent (1). Its pathological features are characterized by excessive colonic inflammation and compromised intestinal mucosal barrier integrity, often leading to disease recurrence even after mucosal healing (2). Although the precise etiology of UC remains incompletely understood, reported pathogenic mechanisms include genetic susceptibility (3), dietary factors (4), immune dysregulation (5), and gut microbiota disturbances (6). The clinical utility of existing treatments is limited by insufficient efficacy and undesirable side effects, highlighting an urgent need for novel therapeutic strategies with improved safety and efficacy profiles.

As a complex chronic condition with significant long-term complications, UC remains a major focus of scientific investigation (7). The clinical translation of multi-omics approaches has been driven by advances in high-throughput sequencing and metabolomics technologies (8). For instance, Sun et al. identified sphingomyelin 1-phosphate as a potential therapeutic target for UC through integrated fecal and plasma metabolomic profiling (9). Supporting evidence from Zhang et al., garnered through metabolomics, indicates that in colitis models, polysaccharides extracted from Holothuria leucospilota adjust several key processes: amino acid metabolism, the production of antimicrobial peptides, and energy homeostasis (10).

Beyond metabolic alterations, gut dysbiosis represents another crucial pathogenic factor in UC (11). While the gut microbiota is essential for maintaining intestinal homeostasis, its disruption can contribute to the development and persistence of mucosal inflammation (12). Advanced microbiome analyses have provided deeper insights into these complex host-microbe interactions. Several beneficial gut species generate short-chain fatty acids (SCFAs). In particular, *Faecalibacterium prausnitzii* produces butyrate, a metabolite which acts as a primary energy source for colonocytes and also confers potent anti-inflammatory effects (13). Notably, *F. prausnitzii* and *Akkermansia muciniphila* have shown synergistic activity in ameliorating chronic inflammatory conditions (14).

Accumulating evidence supports the therapeutic potential of traditional Chinese medicine (TCM) in UC. Several plant-derived compounds have demonstrated efficacy in modulating intestinal inflammation. For instance, *Angelica sinensis* polysaccharides exhibit notable anti-inflammatory activity on colonic epithelial cells. Zou et al. further demonstrated that polysaccharides from the aerial parts of *A. sinensis* significantly attenuate DSS-induced colitis and reduce colonic inflammation (15). Similarly, *Atractylodes macrocephala* alleviates UC-associated gastrointestinal symptoms, including appetite loss, abdominal distension, and diarrhea (16), while oligosaccharides from *Bletilla striata* suppress intestinal inflammation and inhibit pro-inflammatory cytokine release (17). These findings collectively suggest that plant-derived natural products can ameliorate inflammatory bowel disease through gut microbiota modulation and host metabolism regulation (18). *P. notoginseng*, another prominent TCM, demonstrates multiple pharmacological properties, including hematinic, hemostatic, anti-inflammatory, and antioxidant activities (19). Nevertheless, the specific mechanisms through which *P. notoginseng* extract exerts its anti-UC effects remain inadequately explored, particularly regarding its molecular targets and pathway regulation.

To assess the influence of *P. notoginseng* extract on the development of ulcerative colitis, a corresponding mouse model was generated in our research. The key metabolites and differential microorganisms were identified. Through integrated multi-omics analysis, we have uncovered the regulatory network through which a *P. notoginseng* extract treats ulcerative colitis. Specifically, our findings elucidate how this network mediates interactions along the gut microbiota-host metabolism-gut barrier axis.

## 2 Materials and methods

### 2.1 Preparation of *P. notoginseng* extract

The dried rhizomes of *P. notoginseng* were acquired from a commercial supplier (Shenzhen Yiyang Biomedical Technology Co., Ltd., Shenzhen, China). The initial processing involved pulverizing the raw herb and sieving it through a No. 40 mesh. This extraction procedure was carried out in three sequential cycles, each lasting two hours, utilizing a water-to-material ratio maintained at 4:3:3 across the cycles. The three extracts were combined and filtered through a 200-mesh cloth to remove residues. The filtrate was concentrated under vacuum at 60–65°C and -0.08 MPa to achieve a specific gravity of 1.10–1.15 (at 50°C). Subsequently, the concentrated extract was spray-dried with an inlet temperature of 180 ± 5°C and an outlet temperature of 85 ± 5°C to obtain the final extract powder.

To ensure a consistent total saponin concentration of 98% (w/w), multiple extract batches were combined after the key saponinsationrialTA phal R1, ginsenoside Rg1, and ginsenoside Rb1seno been measured via HPLC.

### 2.2 LC-MS-based phytochemical characterization of the extract

Liquid chromatography-mass spectrometry (LC-MS) was utilized to characterize the chemical composition of the *P. notoginseng* extract. The separation was performed on a Waters BEH C18 column (2.1 × 100 mm, 1.7 µm) using a mobile phase that consisted of (A) 0.1% aqueous formic acid and (B) acetonitrile, eluted at 0.3 mL/min, with a 5 µL injection volume. For MS detection, the sheath gas was set to 350 °5 and 12 L/min, and the mass scan range was m/z 50zge,io Data were acquired in both ESI+ (4000 V) and ESI- (3200 V) modes.

### 2.3 Animals and experimental design

Prior to initiation, all studies involving mice were reviewed and authorized by the Biomedical Research Ethics Committee of Hunan Agricultural University (Reference ID: 2024-104). We utilized thirty-two female ICR mice at six weeks of age, which were procured from Hunan Slack Jingda Laboratory Animal Co., Ltd. (License: SCXK (Xiang) 2019-0004) and confirmed to be specific pathogen-free (SPF). The housing conditions for the animals adhered to strict SPF standards, including a controlled 12-hour light/dark cycle, room temperature maintained at 22 ± 1°C, relative humidity at 50%–60%, and free access to both food and water throughout the study.

Following one week of acclimatization, the mice were divided at random into four experimental cohorts, each consisting of eight animals: the normal control (CON), the normal control supplemented with *P. notoginseng* extract (CONSQ), the DSS-induced colitis model (DSS), and the DSS model treated with the extract (DSSSQ).From days 1 to 7, 3% (w/v) dextran sulfate sodium (DSS; Dalian Meilun Biotechnology Co., Ltd., Dalian, China) was administered in the drinking water to the DSS and DSSSQ groups to induce colitis, while the CON and CONSQ groups received normal water. From days 8 to 14, either normal saline (CON and DSS groups) or 200 mg/kg *P. notoginseng* extract in an aqueous solution (CONSQ and DSSSQ groups) was administered by daily oral gavage (0.2 mL/mouse). The disease activity index (DAI) was calculated based on daily records of body weight, stool consistency, and the presence of fecal blood. Following the 14-day experimental period, animals were fasted overnight. After intraperitoneal injection of 100mg/kg of pentobarbital sodium, orbital blood was collected, and the neck was dislocated and killed. Subsequent sample collection included whole blood, colonic segments, fecal contents, and splenic tissue for further examinations.

### 2.4 DAI assessment

Daily assessment of the disease activity index (DAI) followed an established scoring protocol, slightly adapted from prior literature, which integrated three parameters: body weight reduction, stool texture, and presence of fecal blood. The specific criteria for each were:

Reduction in Body Weight (%): 0 (nil), 1 (1il),i 2 (6il),io 3 (11l),ion 4 (exceeding 15%).

Stool Texture: 0 (formed pellets), 2 (soft, loose stool), 4 (watery diarrhea).

Rectal Bleeding: 0 (absent), 2 (occult blood detected), 4 (visible bleeding).

The final DAI value was derived by averaging the scores from these three components, yielding a potential scale of 0 to 12, where elevated values correspond to worsened colonic inflammation.

A commercially available fecal occult blood test kit employing the O-toluidine principle was used for detection. Fresh stool specimens were treated with O-toluidine reagent and an oxidizing agent, followed by monitoring for any color shift within a 120-second window. The outcome was classified from negative to very strongly positive (4+) according to the rapidity and depth of the resulting blue hue, referencing a published guide (20).

### 2.5 Sample collection and histopathological analysis

The index reflecting splenic size was determined by dividing the organ’s fresh weight (in milligrams) by the mouse’s terminal body mass (in grams). To assess colon morphology, samples were first immersion-fixed in 4% paraformaldehyde solution for one day. Subsequent processing involved progressive ethanol dehydration, infiltration with paraffin wax, and microtome sectioning. Slices of 4 µm thickness were subjected to H&E staining, and the resulting slides were digitally scanned for subsequent morphological evaluation.

### 2.6 Immunofluorescence staining

Immunofluorescence staining was employed to evaluate the distribution and expression levels of key intestinal barrier proteins. Deparaffinized and rehydrated colon sections first underwent antigen retrieval. To minimize background, sections were treated with 3% bovine serum albumin (BSA) for blocking. anti-MUC-2 (1:1000, Mitaka, 27675-1-AP), anti-Occludin (1:1000, Mitaka, 27260-1-AP), anti-ZO-1 (1:1000, Mitaka, 27773-1-AP), and anti-claudin-1 (1:1000, Mitaka, 28674-1-AP). Following thorough washes, the sections were exposed to fluorophore-conjugated secondary antibodies for one hour at ambient temperature. Cell nuclei were visualized with DAPI staining, and finally, a confocal laser scanning microscope was used to acquire the fluorescent images.

### 2.7 Analysis of gut microbiota composition via 16S rRNA gene sequencing

Genomic DNA was isolated from fecal contents harvested from the colon and preserved at -80°0. The integrity and concentration of the purified DNA were verified through multiple methods: measurement on a Nanodrop spectrophotometer, quantification with a Qubit fluorometer, and evaluation by electrophoresis on a 0.35% agarose gel. Qualified DNA underwent shearing via ultrasonication, followed by size fractionation employing a BluePippin apparatus. Library preparation was performed with the PNK-LSK109 kit, encompassing steps for repairing DNA ends, adding A-overhangs, and ligating sequencing adapters. The final libraries were then amplified via PCR, their concentration determined again by Qubit, and sequenced on a high-throughput Illumina sequencer.

Raw sequencing data were processed using the fastp software to obtain high-quality clean tags. Alpha diversity was assessed using Shannon, ACE, and Chao1 indices, and beta diversity was analyzed through principal coordinate analysis (PCoA). Based on taxonomic classification, species composition bar plots and bacterial abundance heatmaps were generated at different taxonomic levels. The sequencing accession number of this study can be found at NCBI https://www.ncbi.nlm.nih.gov/bioproject under the BioProject number PRJNA1336462.

### 2.8 Fecal metabolomic profiling

Fecal specimens were subjected to LC-MS-based untargeted metabolomics. In the sample preparation stage, precisely 50 mg of feces was combined with 1 mL of a methanolic/acetonitrile/water (2:2:1, v/v/v) extraction solution that included a 20 mg/L internal standard. The mixtures were vortexed briefly (30 s) and subsequently homogenized through a combination of mechanical grinding and sonication. A pooled quality control (QC) sample was generated by combining aliquots of all individual extracts, and this QC pool was then injected into the LC-MS system for analysis.

### 2.9 Data analysis

All experimental results are presented as the mean ± standard deviation (Mean ± SD). One-way ANOVA was applied to assess differences among more than two groups, while comparisons between two specific groups were carried out with a t-test in GraphPad Prism 9. A probability value (*P*) of less than 0.05 was defined as denoting statistical significance. The IBM SPSS Statistics 25 package was utilized for statistical evaluations. For datasets conforming to a normal distribution, inter-group comparisons across multiple time points were conducted via repeated-measures ANOVA. Image stitching uses Adobe Illustrator, and the raw data has been stored in the National Biotechnology Information Center PRJNA1336462 the BioProject project number.

## 3 Results

### 3.1 LC-MS detection and analysis of *P. notoginseng* extract

**Figure 1:**
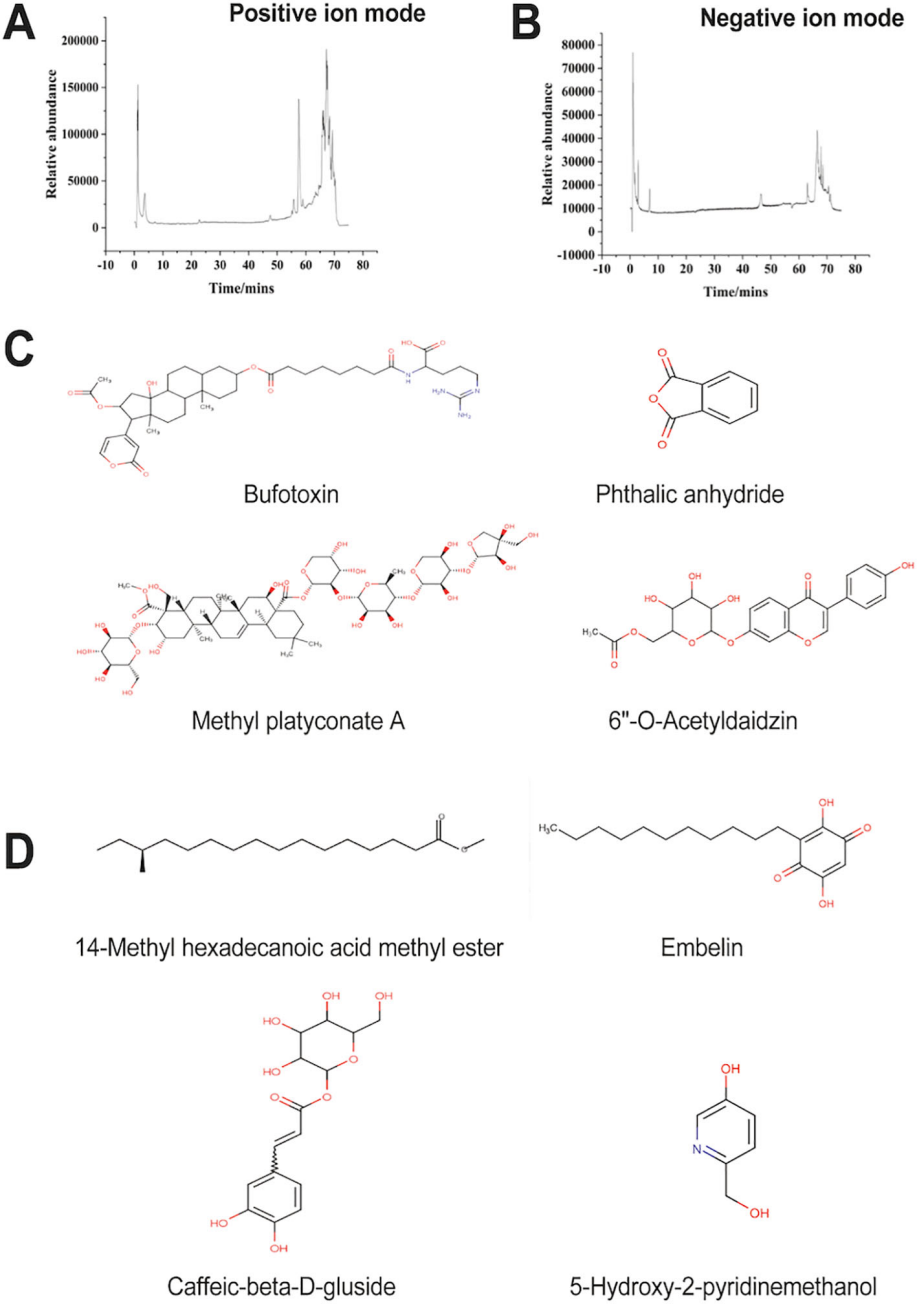
Total ion chromatogram (TIC) of *P. notoginseng* extract and the top 4 chemical composition structural formulas **(A)** Total ion chromatogram (TIC) under positive electrospray ionization, **(B)** TIC under negative electrospray ionization, **(C)** Top 4 chemical composition formulas of *notoginseng* extract scored in positive ion mode, **(D)** The top 4 chemical composition structural formulas of notoginseng extract scored in negative ion mode.

LC-MS analysis characterized the chemical profile of the *P. notoginseng* extract. **Figures 1A, B** displays the corresponding total ion chromatograms (TICs) from the positive and negative ionization modes. By matching the Chinese herbal medicine database, the top 20 chemical components were screened out (**Tables 1**, **2**), and the chemical formulas of the top 4 were shown in **Figures 1C, D**. Among them, in positive ion mode, the top four chemical components are Bufotoxin (C_40_H_60_N_4_O_10_), Phthalic anhydride (C_8_H_4_O_3_), Methyl platyconate A (C_58_H_92_O_29_), 6”-O-Acetyldaidzin (C_23_H_22_O_10_). The top four chemical components in negative ion mode are: 14-Methyl hexadecanoic acid methyl ester (C_18_H_36_O_2_), Embelin(C_17_H_26_O_4_), Caffeic-beta-D-gluside (C_15_H_18_O_9_), 5-Hydroxy-2-pyridinemethanol (C_6_H_7_NO_2_).

**Table 1:** Top 20 chemical constituents of *Panax notoginseng* extract scores identified in positive ion mode.

Number	Score	Area	Name	RT	Formula	Mass
1	99.05	52547	Bufotoxin	10.252	C40H60N4O10	756.4315
2	99	74375	Phthalic anhydride	69.979	C8H4O3	148.0164
3	98.7	379453	Methyl platyconate A	10.252	C58H92O29	1252.573
4	98.49	218268	6”-O-Acetyldaidzin	6.58	C23H22O10	458.1205
5	98.49	115529	Cinobufotoxin	5.559	C40H58N4O10	754.4159
6	98.44	28979	L-(-)-alpha-Monopalmitin	1.183	C19H38O4	330.2774
7	97.68	15767340	5-Hydroxy-2-pyridinemethanol	1.587	C6H7NO2	125.0482
8	97.66	25056	3beta,15xi,16-Trihydroxy isopimaric acid	31.493	C20H32O5	352.2241
9	97.63	18324	Kudinoside B	24.227	C47H74O19	942.4818
10	97.44	4131298	Betaine	1.048	C5H11NO2	117.0796
11	97.32	55112	Andrographolide	26.427	C20H30O5	350.2087
12	97.16	828205	Ugonin B	58.959	C26H28O6	436.1877
13	97.01	53792	Hovenine A	3.57	C27H42N4O4	486.3198
14	96.93	138586	3-O-beta-D-Xylopyranosyl-esculentic acid	24.344	C35H54O10	634.3708
15	96.87	47819	Camellidin II	23.194	C53H84O24	1104.534
16	96.85	1296392	Andrographolide	25.304	C20H30O5	350.2086
17	96.77	181630	Glycyrol	0.913	C21H18O6	366.1109
18	96.76	15320	Betaine	73.543	C5H11NO2	117.0792
19	96.5	8533	Rhododendrin	49.463	C16H24O7	328.1503
20	96.47	211241	Agavoside A	21.731	C33H52O9	592.3626

**Table 2:** Top 20 chemical constituents of *Panax notoginseng* extract scores identified in negative ion mode.

Number	Score	Area	Name	RT	Formula	Mass
1	99.91	2602756	14-Methyl hexadecanoic acid methyl ester	68.505	C18H36O2	284.2715
2	99.73	6638952	Embelin	46.515	C17H26O4	294.1829
3	99.72	850512	Caffeic-beta-D-gluside	2.429	C15H18O9	342.0949
4	99.63	1446456	5-Hydroxy-2-pyridinemethanol	1.543	C6H7NO2	125.0478
5	99.5	285549	Methyl(2,4-dihydroxy-3-formyl-6-methoxy) phenylketone	2.193	C10H10O5	210.0528
6	99.39	82103	Gluconic acid	3.046	C6H12O7	196.0584
7	99.31	825184	2,3-Dihydro-5,7-dihydroxy-2,6,8-trimethyl-4H-1-benzopyran-4-one	23.23	C12H14O4	222.0889
8	99.21	216812	Plumbagin	54.559	C11H8O3	188.047
9	98.75	58354	Plumbagin	65.746	C11H8O3	188.047
10	98.75	168523	Chrysotoxine	58.783	C18H22O5	318.1463
11	98.7	40767	Methylanthranilate	3.29	C8H9NO2	151.0634
12	98.67	31140	Citric acid	0.56	C6H8O7	192.0273
13	98.36	3382811	12-Methyl tetradecanoic acid methyl ester	67.819	C16H32O2	256.2403
14	98.24	284697	Embelin	57.486	C17H26O4	294.1824
15	98.21	230993	Dibutyl phthalate	63.494	C16H22O4	278.1513
16	98.03	1414828	2-(3,4-Methylenedioxyphenyl)-3-methyl-5-(2-oxopropyl) benzofuran	54.256	C19H16O4	308.1042
17	97.74	211588	(-)2D,4D,6D,8D-Tetramethyl undecanoic acid	67.574	C15H30O2	242.224
18	97.58	591941	Utendin	66.891	C21H34O5	366.2401
19	97.54	1675566	Asarumin B	46.524	C13H16O4	236.1046
20	97.46	139418	2,4,4’-Trihydroxychalcone	46.574	C23H24O12	492.1266

### 3.2 Administration of *P. notoginseng* extract significantly ameliorated disease severity in UC mice, as evidenced by improved body weight loss, bloody stools, and diarrhea

**Figure 2:**
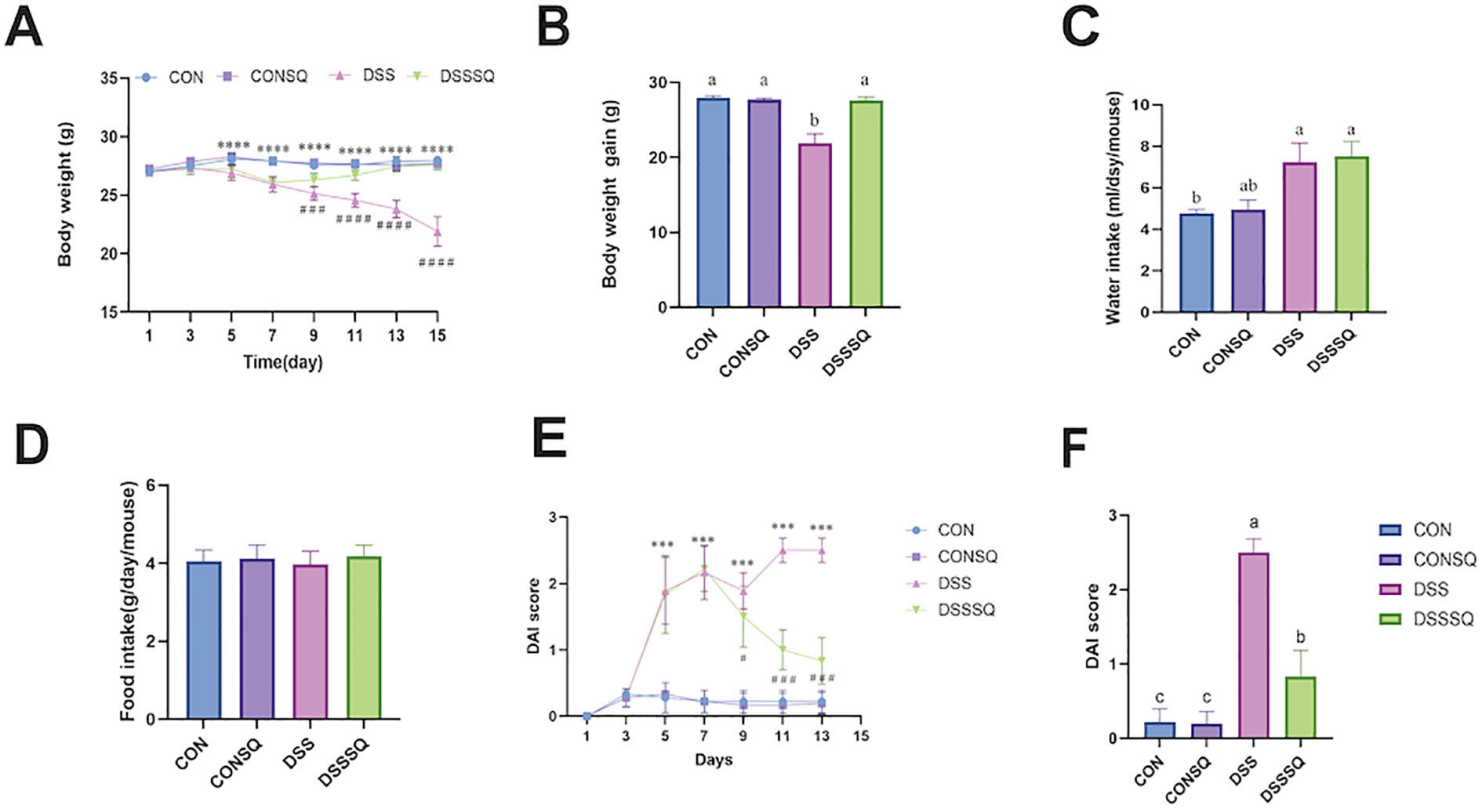
Effects of *P. notoginseng* extract on physiological parameters in a murine colitis model. **(A)** Body weight dynamics over the 14-day experimental period. **(B)** Net body weight change at the endpoint. **(C)** Daily water consumption. **(D)** Daily food intake. **(E)** Disease activity index (DAI) scores recorded throughout the study. **(F)** Final DAI scores at termination. Symbols denote specific group comparisons: "*" CON vs. DSS; "#" DSS vs. DSSSQ. Asterisks and number signs indicate statistical significance levels: # P < 0.05, ***/### P < 0.001, ****/#### P < 0.0001. In panels B and F, distinct lowercase letters (a, b, c) indicate significant intergroup differences (P < 0.05), while shared letters denote non-significant differences (P > 0.05).

Body weight remained stable over the course of the experiment in the control (CON) mice. Oppositely, DSS-exposed animals developed characteristic clinical symptoms like diminished locomotion, poor coat condition, and considerable loss of body mass. Graphical data in **Figure 2A** demonstrate a statistically noteworthy drop in the body weight of the DSS group relative to the CON group, beginning on day 5 (*P* < 0.05). Treatment with *P. notoginseng* extract in the DSSSQ group significantly alleviated this DSS-provoked weight reduction, resulting in a higher terminal body weight compared to the untreated DSS model (*P* < 0.05). At the experiment’s conclusion (**Figure 2B**), body weight was minimal in the DSS group, but the extract-administered DSSSQ group recovered to a mass comparable to the normal control group (*P* < 0.05). Analysis of fluid intake (**Figure 2C**) revealed elevated daily water consumption in the DSS and DSSSQ groups relative to both the CON and CONSQ groups (*P* < 0.05). However, no significant variations were observed between groups regarding mean daily food intake (**Figure 2D**). Therefore, the results substantiate that *P. notoginseng* extract effectively attenuates body weight loss linked to UC in mice.

DAI serves as a crucial indicator for evaluating the severity of colitis in murine models. After administration of DSS, initial clinical manifestations such as diarrhea and fecal bleeding started to appear around day 4. Data presented in **Figure 2E** reveal that from day 5, the DAI values for the DSS cohort were considerably elevated relative to the control group (*P* < 0.05). A key observation is that, beginning on day 9, the DAI scores of the DSSSQ animals dropped significantly below those of the untreated DSS model group (*P* < 0.05). Moreover, a consistent downward trend in the DAI was noted in the DSSSQ group from day 7 onwards, with values eventually nearing those recorded in the CON and CONSQ cohorts. At the study’s conclusion (**Figure 2F**), the DSS group continued to display the highest DAI, significantly exceeding the scores of both control groups. Conversely, the DSSSQ group’s final DAI was substantially lower than the DSS group’s (*P* < 0.05) and was statistically indistinguishable from the CON group. These collective results demonstrate that treatment with P. notoginseng extract successfully mitigates clinical symptoms like diarrhea and intestinal bleeding provoked by DSS in mice.

### 3.3 Protective effect of PN on colon length, spleen index and intestinal barrier in mice with colitis

**Figure 3:**
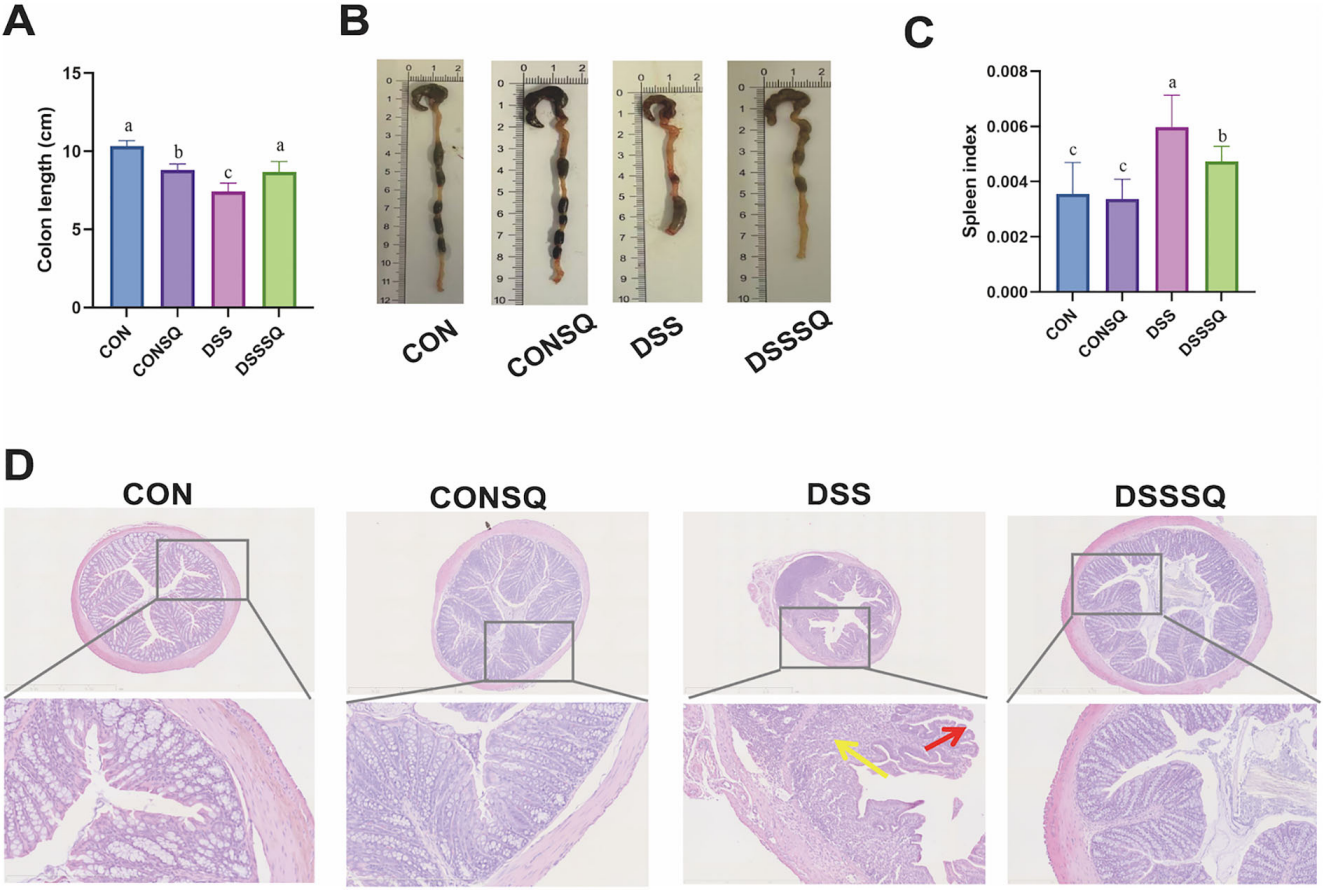
Macroscopic and histological assessments of colitis severity. **(A)** Statistical summary of colon length measurements. **(B)** Visual appearance of excised colons. **(C)** Spleen index across groups. **(D)** Representative H&E-stained colon sections (scale bar: 200 μm). In bar graphs **(A, C)**, groups labeled with different letters differ significantly (*P < 0.05*); groups sharing a common letter are not significantly different (*P > 0.05*).

DSS-induced murine colitis is characterized by intensified gut inflammation, intestinal spasms, mucosal thinning, villus destruction, and colonic shortening. Representative data in **Figures 3A, B** indicate a marked reduction in colon length in the DSS cohort relative to all other groups (*P* < 0.05). Administration of *P. notoginseng* extract normalized this parameter in the DSSSQ group, bringing it back to a typical range. The spleen index, a valuable indicator of systemic inflammatory status, often becomes elevated following DSS challenge, potentially reflecting immune dysregulation. As depicted in **Figure 3C**, a significant increase in the spleen index was found in the DSS group compared to the CON group (*P* < 0.01), indicative of splenomegaly. This increase was substantially reversed by *P. notoginseng* extract treatment in the DSSSQ group, with values approaching those seen in normal mice. In summary, oral administration of the aqueous *P. notoginseng* extract partially reversed colonic shortening and mitigated spleen enlargement in the UC mouse model.

By performing HE staining analysis on mouse colon tissue (**Figure 3D**), it can be seen that the colon morphology and structure of the mice in the CON and CONSQ groups are normal, and there is no inflammatory infiltration or pathological damage. Inflammatory infiltrates (red arrows) and colonic villus destruction (yellow arrows) in the DSS group. Both conditions improved significantly in the DSSSQ group. These results highlight the potential of *P. notoginseng* extract in mitigating inflammatory damage caused by UC.

### 3.4 *P. notoginseng* extract protects the intestinal barrier via upregulation of tight junction proteins and MUC-2

**Figure 4:**
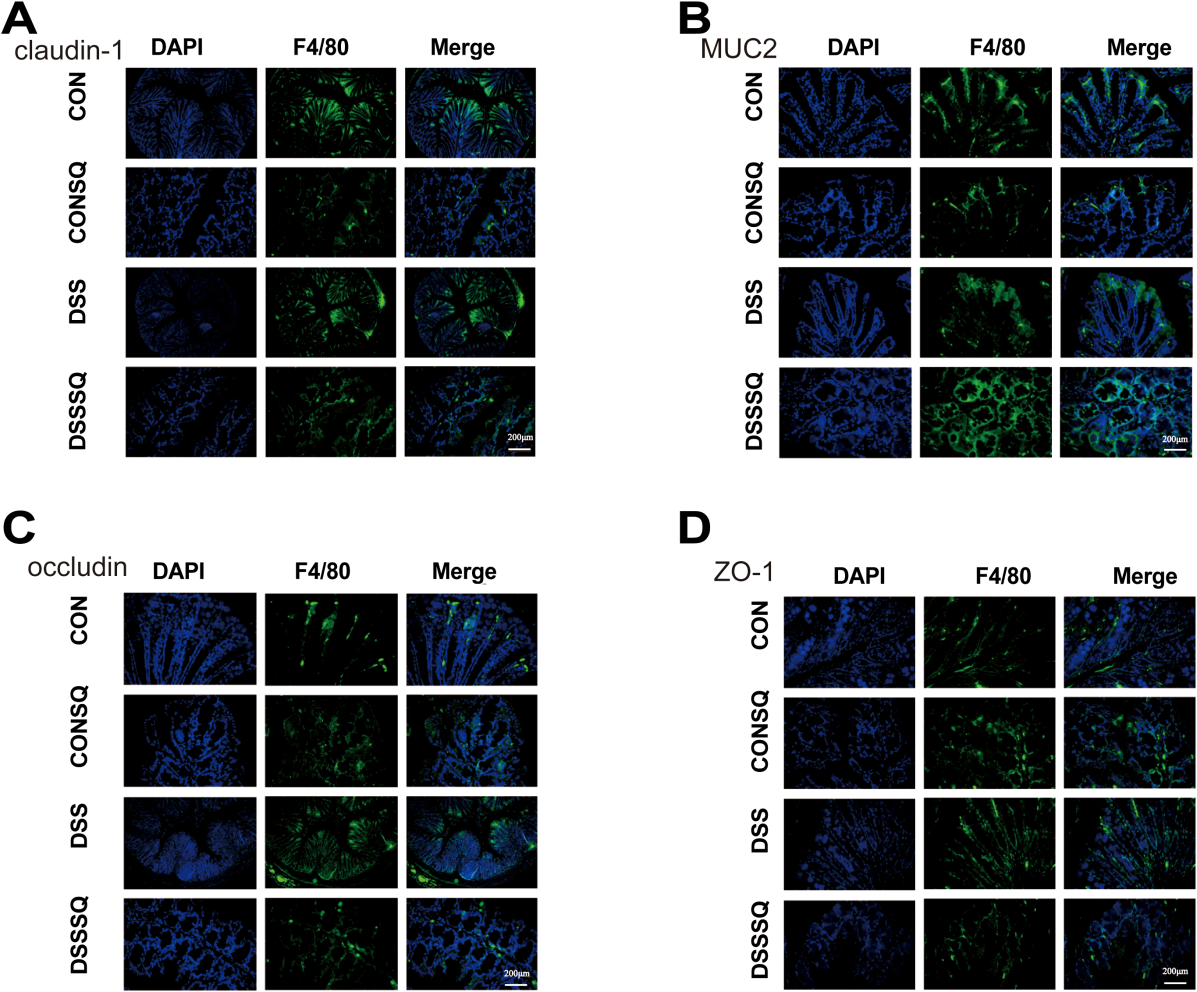
Representative immunofluorescence images. **(A)** Representative immunofluorescence images showing *in situ* expression of claudin-1 protein. **(B)** Representative immunofluorescence images showing *in situ* expression of MUC2 protein. **(C)** Representative immunofluorescence images showing *in situ* expression of occludin protein. **(D)** Representative immunofluorescence images showing *in situ* expression of ZO-1 protein. Scale bar = 200μm. Data are expressed as mean ± standard deviation. (n = 6).

To assess the integrity of the intestinal epithelium, we analyzed the expression levels of the tight junction (TJ) proteins MUC-2 and mucin Claudin-1, Occludin, and ZO-1. As shown in **Figure 4**, all indicators in the CON group (MUC2, Occludin, Claudin-1, ZO-1): were at the highest level in the histogram, and the light image showed a bright, continuous, and complete signal. Compared with CON, the expression of TJ proteins Occludin, Claudin-1, and ZO-1 in the DSS group was greatly reduced, the function of goblet cells was impaired, the mucus layer became thinner or disappeared, the chemical barrier collapsed, and epithelial cells were directly exposed to harmful substances, as evidenced by the results of immunofluorescence staining. There was no significant difference in all indicators of CONSQ compared with the CON group, which once again confirmed that *P. notoginseng* extract itself had no adverse effect on the normal intestinal barrier.

At the same time, DSSSQ was observed to upregulate or stabilize the expression of tight junction proteins, promote goblet cell regeneration, repair the damaged intestinal physical barrier, and reduce macrophage infiltration, thereby effectively alleviating intestinal inflammation. It acts simultaneously on multiple key proteins of the mucus barrier and tight junction barrier, leading to comprehensive restoration of intestinal barrier function. Therefore, both qualitative and quantitative analyses indicated that *P. notoginseng* extract significantly reversed DSS-induced barrier damage.

### 3.5 *P. notoginseng* extract remodeled the intestinal microbiota structure of colitis mice

**Figure 5:**
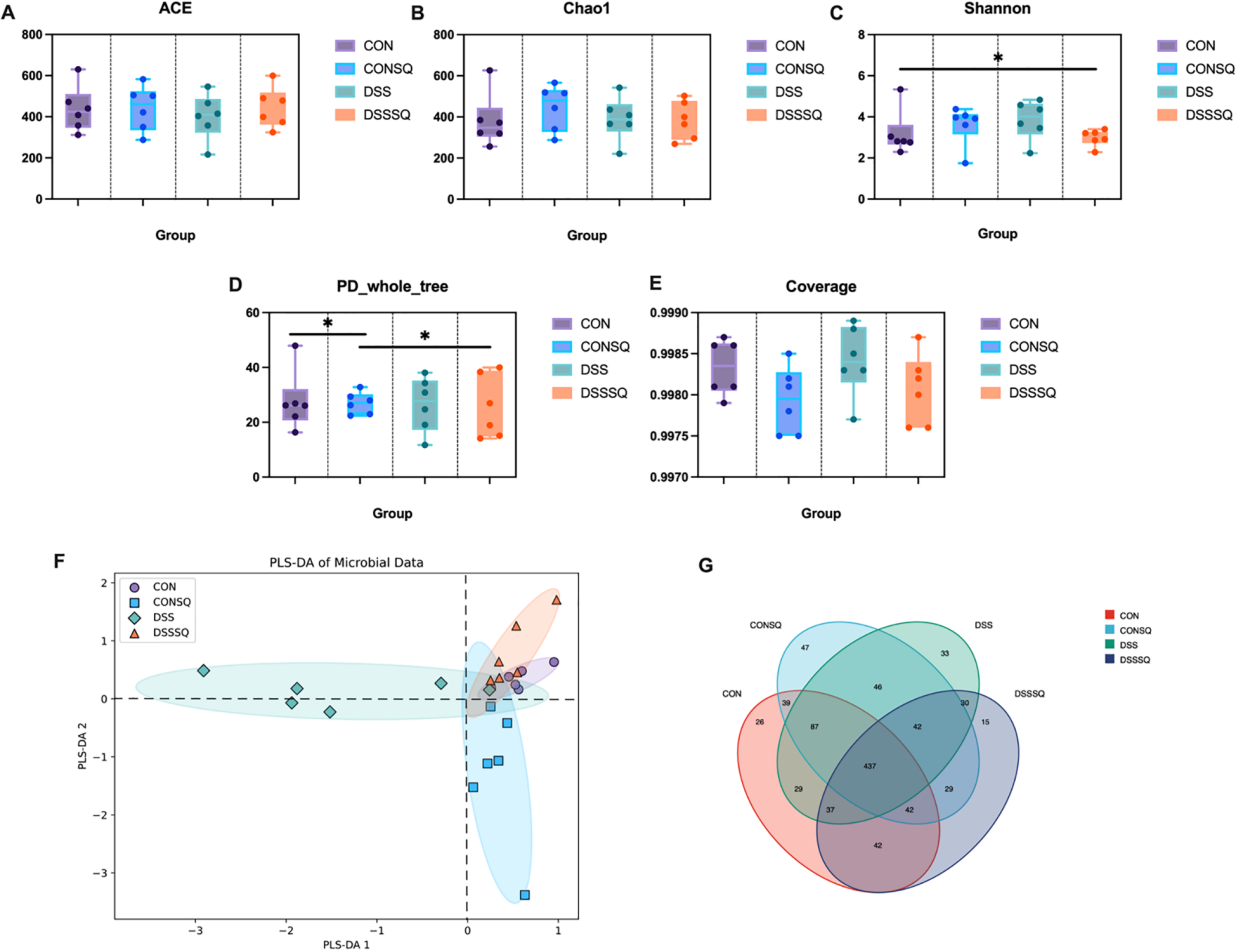
Assessment of intestinal microbiota structure and composition. **(A–E)** Alpha diversity metrics, including ACE, Chao1, Shannon, PD_whole_tree, and Coverage indices. **(F)** PLS-DA visualization for beta diversity. **(G)** Venn diagram (Wayne diagram) showing group-specific and common OTUs. * P < 0.05.

Alpha diversity of the gut microbiota was assessed using several indices, including ACE, Chao1, Shannon, PD_whole_tree, and Coverage. While the DSS group exhibited a decreasing trend in the ACE index relative to the other three cohorts, this difference did not reach statistical significance (**Figure 5A**). For the Chao1 and Shannon indices, comparisons between the CON and DSS groups, as well as between the DSS and DSSSQ groups, revealed no statistically significant differences, though the values in the DSS group were numerically elevated compared to both the CON and DSSSQ groups. This observation could potentially be attributed to microbial community imbalance and selective proliferation of certain beneficial bacteria following DSS challenge (**Figures 5B, C**). Analysis of PD_whole_tree values uncovered significant differences between the CON and CONSQ groups, and between the CONSQ and DSSSQ groups (*P* < 0.05), implying that administration of *P. notoginseng* extract influences the phylogenetic diversity and evolutionary trajectory of the gut microbial ecosystem, thereby altering its community structure (**Figure 5D**). Finally, the Coverage values across all four groups exceeded 99.7%, with no significant inter-group disparities observed. This high coverage rate confirms adequate sequencing depth, comprehensive data representation, and high confidence in the sequencing results (**Figure 5E**).

The beta diversity results were analyzed and a PLS-DA plot was plotted (**Figure 5F**). From the perspective of PLS-DA1, with 0 as the boundary, it can be seen that most of the DSS group is concentrated on the left side of the cut-off line, and the CON, CONSQ and DSSSQ groups are concentrated on the right side of the cut-off line, indicating that the intestinal microbiota structure of the DSS group has significant cluster differences with other groups, and also shows that the mice with enteritis disease are also closer to homeostasis under the treatment of *P. notoginseng*.

The results of the Venn diagram showed (**Figure 5G**) that different treatments had a significant effect on the structure of the gut microbial community, among which the CONSQ group was outstanding in maintaining or increasing microbial diversity, while the DSSSQ group had fewer endemic species, which may reflect the regulatory effect on the microbiota structure under the enteritis disease model of *P. notoginseng* intervention.

### 3.6 Structural composition of intestinal flora in mouse enteritis model under *P. notoginseng* intervention

**Figure 6:**
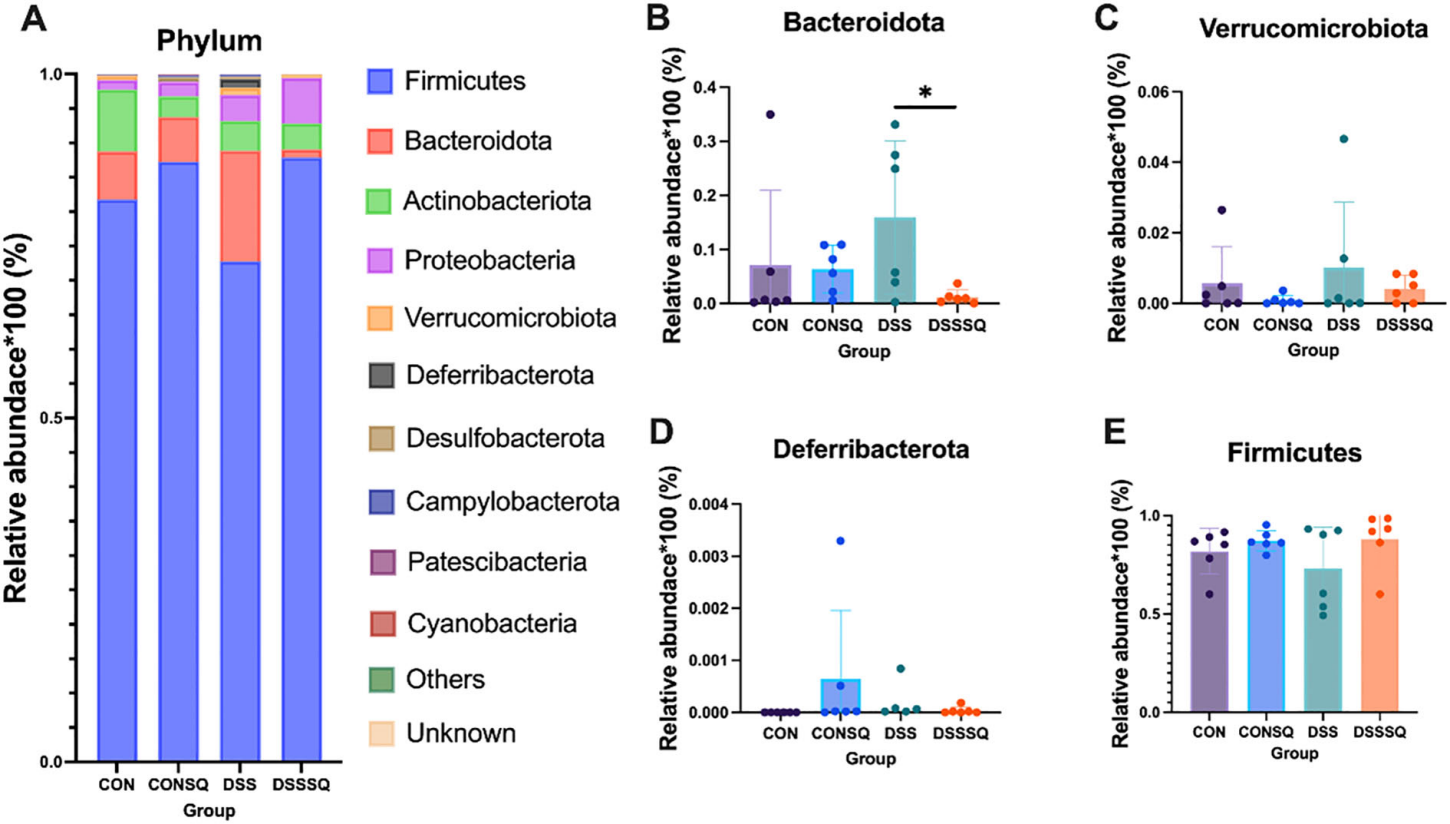
Structure of intestinal flora at the Phylum level. **(A)** Top 10 population abundance in mouse colonic contents, **(B)** Bacteroidota, **(C)** Verrucomicrobiota, **(D)** Deferribacterota, **(E)** Firmicutes. The results are expressed as mean ± SE *P < 0.05, determined by the LSD-t test.

**Figure 7:**
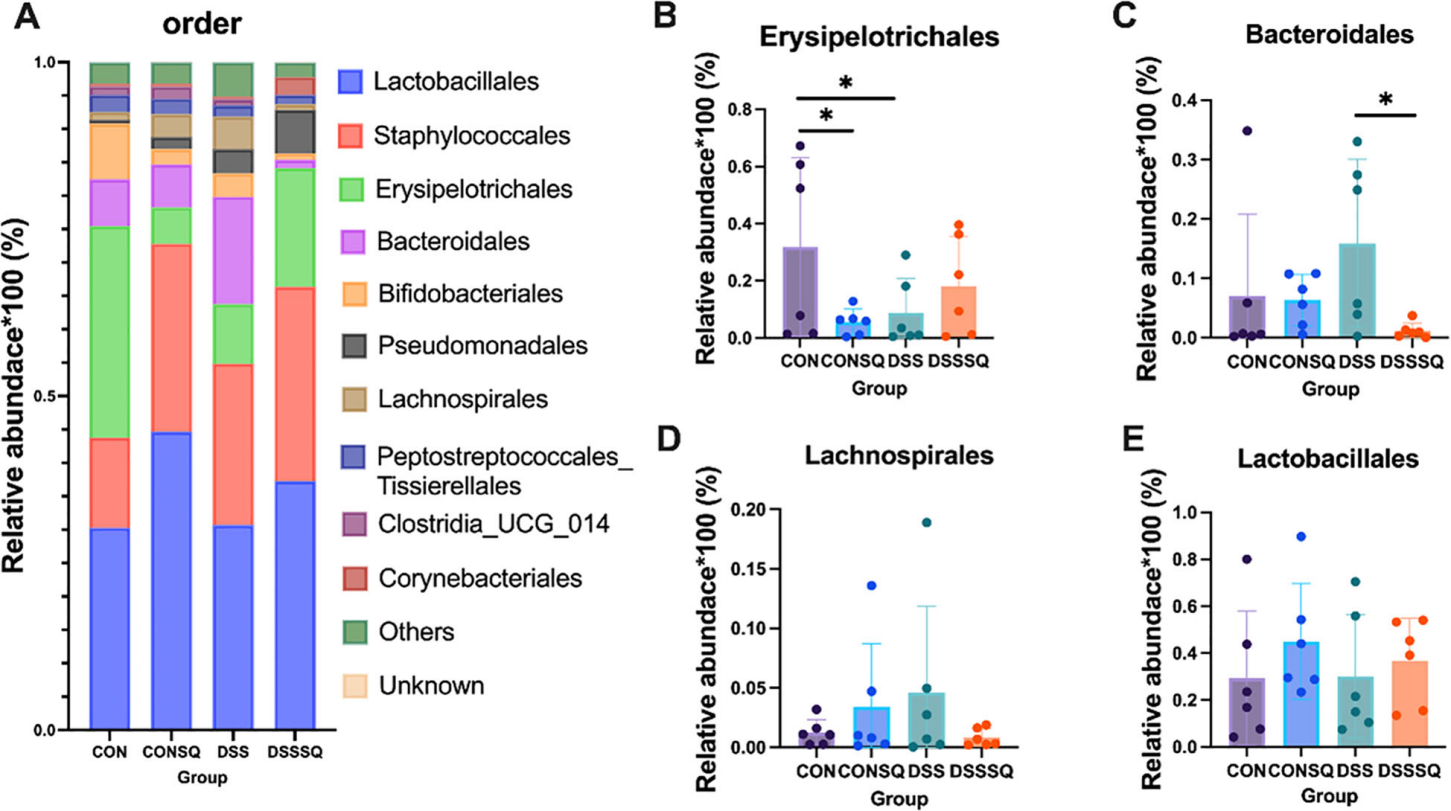
Structure of intestinal flora at the order level. **(A)** Top 10 population abundance in mouse colon contents. **(B)***Erysipelotrichales*; **(C)***Bacteroidales*; **(D)***Lachnospirales*; **(E)***Lactobacillales*. The results are expressed as mean ± SE *P < 0.05, determined by the LSD-t test.

**Figure 8:**
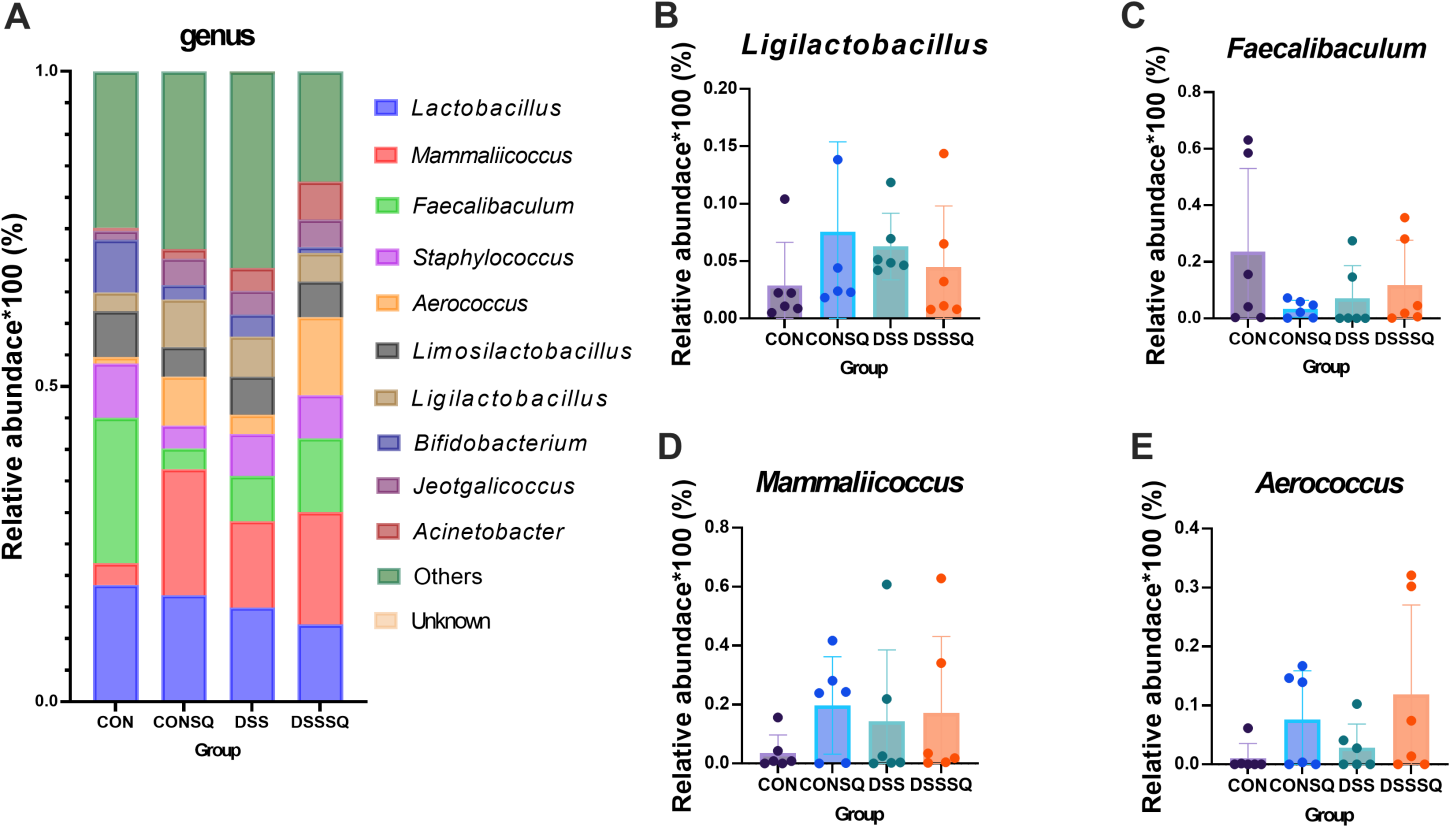
Structure of intestinal flora at the Genus level. **(A)** Top 10 population abundance in mouse colon contents. **(B)***Ligilactobacillus*; **(C)***Faecalibacuclum*; **(D)***Mammaliicoccus*; **(E)***Aerococcus*.

Profiling of the gut microbiota via 16S rRNA sequencing indicated that the onset of colitis coincided with marked perturbations in microbial community structure and population dynamics. These dysbiotic patterns were further delineated through a hierarchical taxonomic examination, as visualized at the phylum, order, and genus levels in **Figures 6**–**8**.

#### 3.6.1 Phylum-level restructuring of the gut microbiota in response to *P. notoginseng* intervention in murine colitis

The top 10 flora at the phylum level were selected for analysis (**Figure 6A**), with Firmicutes, Bacteroidota, Actinobacterioa and Proteobacteria being the top four microorganisms. F/B (Firmicute vs. Bacteroidota) is an important measure of healthy homeostasis of gut microbes. The abundance of Firmicutes decreased in DSS, while the abundance of Firmicutes in enteritis model mice was reversed after treatment with *P. notoginseng*, even higher than that of the CON group (**Figure 6B**). Bacteroidota increased in abundance in the DSS group, and there was a significant reversal in the DSSSQ group compared with the DSSSQ group after *P. notoginseng* treatment (*P* < 0.05) (**Figure 6C**). A notable finding was the elevated Firmicutes and reduced Bacteroidota levels in the DSSSQ group relative to the DSS model, resulting in a Firmicutes/Bacteroidota (F/B) ratio that shifted towards the normal range observed in the CON group. This shift suggests a partial restoration of microbial balance following *P. notoginseng* treatment. Furthermore, the DSS group exhibited an abnormal rise in Verrucomicrobiota, which was attenuated by the extract administration, albeit without statistical significance (**Figure 6D**). In the CONSQ group, the mean abundance of Deferribacterota appeared elevated; however, this was driven by a subset of individuals with high values, while most subjects showed negligible levels. The underlying cause for this heterogeneity remains unknown but could be attributed to inter-individual variation (**Figure 6E**).

#### 3.6.2 Changes in the level of intestinal flora structure in mouse enteritis model under *P. notoginseng* intervention

The structure and composition of the top ten intestinal flora in terms of abundance at the eye level (**Figure 7**). The average abundance of *Erysipelotrichales* in the CON group was very high (**Figure 7A**), while the average abundance in the DSS group decreased significantly (*P<0.05*), while the abundance of *Erysipelotrichales* in the DSSSQ group was reversed. Notably, there was also a significant decrease in the CONSQ group (compared to the CON group) (*P<0.05*), for unknown reasons (**Figure 7B**). The relative abundance of *Bacteroidales* remained stable in both the CON and CONSQ groups, suggesting that *P. notoginseng* extract itself does not directly modulate this taxonomic order under physiological conditions. However, a significant reversal of its abundance was observed in the DSSSQ group compared to the DSS model (*P* < 0.05), demonstrating that the extract restructures the microbial community in colitis (**Figure 7C**). Enteritis induction promoted an increase in *Lachnospirales*, which was counteracted by *P. notoginseng* treatment in the DSSSQ cohort (**Figure 7D**). While *Lactobacillales* levels were largely unaltered in the DSS group, they showed an upward trend in both the CONSQ and DSSSQ groups (**Figure 7E**). This suggests a potential prebiotic-like effect of the extract that favors *Lactobacillales*, even though the inter-group differences did not reach statistical significance.

#### 3.6.3 Changes in the structure of intestinal flora in mouse enteritis model under *P. notoginseng* intervention

The top ten population abundance at the Genus level (**Figure 8A**) are: *Lactobacillus*, *Mammaliicoccus*, *Faecalibacuclum*, *Staphylococcus*, *Aerococcus*, *Limosilactobacillus*, *Ligilactobacillus*, *Bifidobacterium*, *Jeotgalicoccus*, *Acinetobacter*. The abundance of *Ligilactobacillus* was increased in the DSS group (compared with the CON group), but it was remitted in the DSSSQ group after *P. notoginseng* intervention (**Figure 8B**). *Faecalibacuclum* decreased in the DSS group (compared with the CON group) and was reversed after *P. notoginseng* intervention (**Figure 8C**); The changes in the abundance of these two types of bacteria indicate that *P. notoginseng* can indeed improve the intestinal flora structure of enteritis model mice. However, *Mammaliicoccus* and *Aerococcus* both increased under the action of *P. notoginseng* (CONSQ and DSSSQ groups) (**Figures 8D,E**), but neither was statistically significant, probably because *P. notoginseng* had a promoting effect on the growth of these bacteria, and the specific reason was unknown.

#### 3.6.4 Correlation between phylum levels of gut microbiota and heat maps of each level

**Figure 9:**
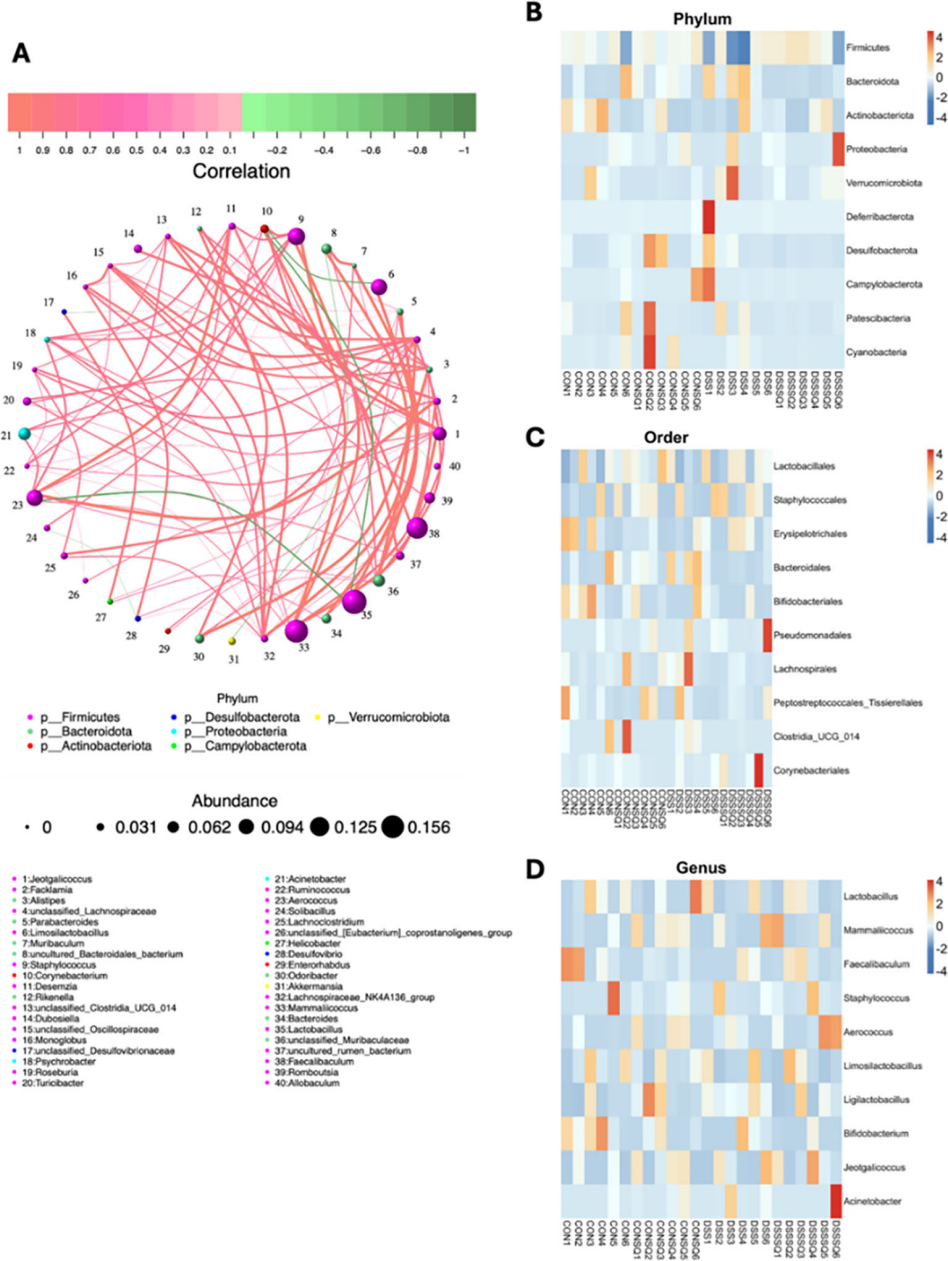
Microbial network correlation and heat maps at each level. **(A)** Correlation between Phylum level gut microbiota, **(B)** Phylum level heat map, **(C)** Order level heat map, **(D)** Genus level heat map.

As shown in **Figure 9A**, there is a very strong correlation between the populations of the main components of the gut microbiota, indicating that changes in the structure of the gut microbiota play a key role in enteritis model mice.

### 3.7 Regulation of *P. notoginseng* extract on metabolic disorders in UC mice

**Figure 10:**
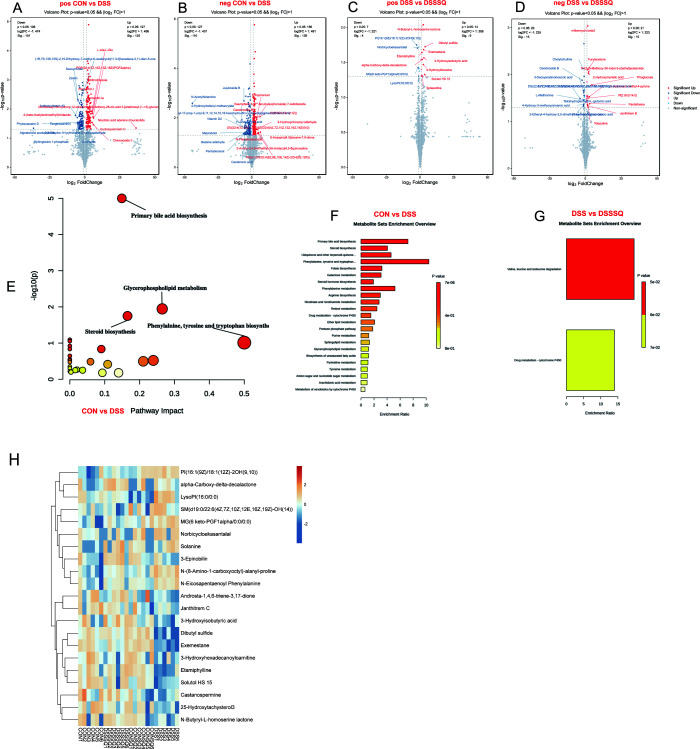
Volcanic cluster composite of mouse fecal metabolites. **(A-D)** Volcanic diagram of metabolite changes in positive/negative ion mode, **(A)** CON group and DSS group in positive ion mode, **(B)** CON group and DSS group in negative ion mode, and **(C)** DSS group and DSSSQ group in positive ion mode, **(D)** Negative ion mode DSS group and DSSSQ group, **(E)** Metabolic pathway influence map, **(F)** Metabolite set enrichment overview map of CON and DSS groups, **(G)** Metabolite set enrichment overview map of DSS and DSSSQ groups, **(H)** Differential metabolite heat map of each treatment group (n=6).

In volcanic map analysis, the results showed that in positive ion mode, 101 metabolite levels were down-regulated and 125 metabolites were up-regulated in the DSS group compared to the CON group (**Figure 10A**). Compared with the DSS group, the levels of 4 metabolites were down-regulated and 9 metabolites were up-regulated (|log2FC|>1, *P<0.05*) (**Figure 10C**). In the negative ion mode, 110 metabolites were down-regulated and 139 metabolites were up-regulated in the DSS group compared to the CON group (**Figure 10B**). Compared with the DSS group, 15 metabolites were down-regulated and 15 metabolites were up-regulated (|log2FC|>1, *P<0.05*) (**Figure 10D**).

Metabolic pathway enrichment analysis revealed that P. notoginseng intervention significantly modulated several biochemical pathways compared to the DSS model. As depicted in **Figure 10E**, these included primary bile acid biosynthesis, glycerophospholipid metabolism, steroid biosynthesis, and the biosynthesis of phenylalanine, tyrosine, and tryptophan, with primary bile acid biosynthesis exhibiting the most pronounced alterations. Further analysis in **Figure 10F** demonstrated distinct enrichment patterns for multiple pathways, including primary bile acid biosynthesis and the urea cycle, between the CON and DSS groups. The associated enrichment ratios and P-values confirmed substantial changes in the metabolic activity of these pathways. Furthermore, comparison between the DSS and DSSSQ groups (**Figure 10G**) identified significant enrichment in valine, leucine, and isoleucine degradation pathways. In contrast, the drug metabolism-cytochrome P450 pathway remained largely unchanged, highlighting a specific regulatory role of the treatment in branched-chain amino acid catabolism.

To identify specific differential metabolites, this study was conducted with | Log2FC|>0.5, *P<0.05*, and VIP>1 was used as the filter conditions, and 21 differential metabolites were screened out, and a differential metabolite heat map was constructed (**Figure 10H**).

As shown in **Figure 10H**, among the 21 differential metabolites, N-(8-Amino-1-carboxyoctyl)-alanyl-prolineLyso, PI (16:0/0:0), PI (16:1(9Z)/18:1(12Z)-2OH (9,10)), N-Eicosapentaenoyl Phenylalanine, and MG compared to the CON group (6 keto-PGF1alpha/0:0/0:0) was significantly up-regulated in the DSS group (*P<0.05*). Compared with the CON group, 25-Hydroxytachysterol3, Exemestane, Castanospermine, 3-Hydroxylidocaine, Etamiphylline, 3-Hydroxylidocaine, Solasodine, Janthitrem C, Solutol HS 15, N-Butyryl-L-homoserine lactone and Dibutyl sulfide were significantly down-regulated in the DSS group (*P<0.05*). Compared with the DSS group, PI (16:1(9Z)/18:1(12Z)-2OH(9,10)), LysoPI (16:0/0:0), N-(8-Amino-1-carboxyoctyl)-alanyl-proline, N-Eicosapentaenoyl Phenylalanine, MG (6 keto-PGF1alpha/0:0/0:0) was significantly down-regulated in the DSSSQ group (*P<0.05*), which was close to the level of the normal group. Compared with the DSS group, alpha-Carboxy-delta-decalactone, Exemestane, 3-Epinobilin, Castanospermine, 3-Hydroxylidocaine, Etamiphylline, 3-Hydroxylidocaine, Solasodine, Janthitrem C, Solutol HS 15, N-Butyryl-L-homoserine lactone and Dibutyl sulfide were significantly up-regulated in the DSSSQ group and close to the level of the CON group. The changes in these metabolite levels are largely consistent with the metabolite heat map. The above results indicate that *P. notoginseng* extract can regulate the level of disordered metabolites to a certain extent, and these significantly changed metabolites may become biomarkers for *P. notoginseng* extract to regulate UC, which can be used to explore the pathological mechanism of UC.

### 3.8 Correlation analysis of metabolomics and microbiome

**Figure 11:**
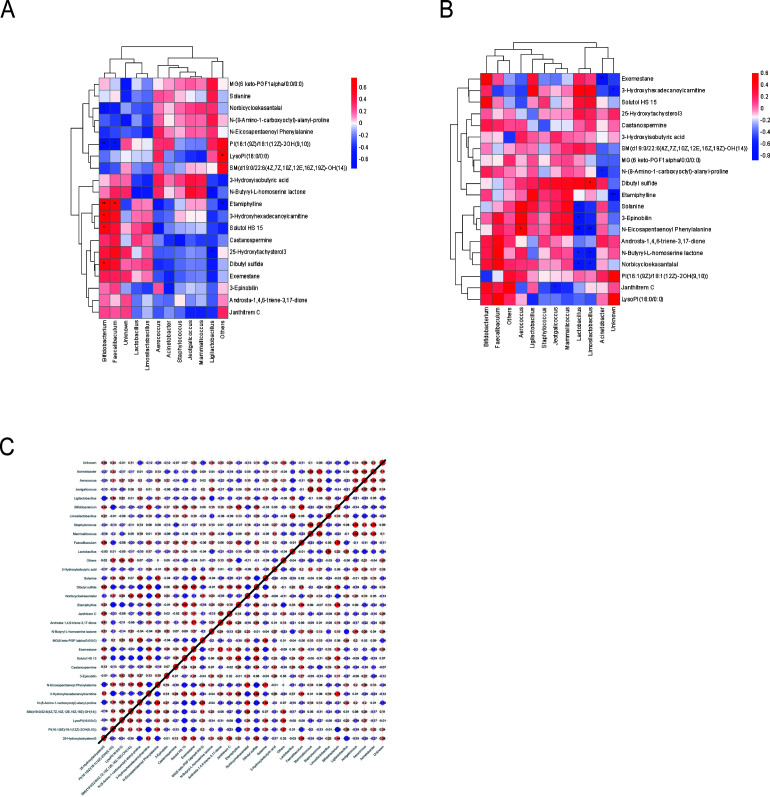
Correlation analysis in metabolomics and microbiome. **(A)** CON VS DSS Correlation Heatmap, **(B)** DSS VS DSSSQ Correlation Heatmap, **(C)** CON VS DSS Critical Correlation Network (top left), DSS VS DSSSQ Critical Correlation Network (bottom right). Red indicates a positive correlation and blue indicates a negative correlation. “*” means *P* < 0.05, “**” means *P* < 0.01, and “***” means *P* < 0001.

Integrated analysis of metabolomic and microbiomic data revealed significant microbiota-metabolite associations, as visualized in **Figure 11**. The correlation heatmap comparing CON and DSS groups (**Figure 11A**) demonstrated a strong positive correlation between Bifidobacterium and Etamiphylline (*P* < 0.01). Additionally, Faecalibaculum abundance was positively linked with Etamiphylline levels (*P* < 0.01), while 3-Hydroxhexadecanolcarnitine, Solutol HS 15, and Dibutyl sulfide showed significant positive correlations with each other (*P* < 0.05). In the DSS versus DSSSQ comparison, 3-Hydroxyisobutyric acid exhibited markedly positive correlations with probiotic genera including *Bifidobacterium*, *Lactobacillus*, and *Limosilactobacillus* (*P* < 0.01). The interaction network depicted in **Figure 11C** effectively integrates and confirms these key relationships identified in the heatmap analyses.

## 4 Discussion

UC pathogenesis involves a highly complex interplay of factors, among which gut microbiota dysregulation has been recognized as a pivotal pathogenic element. To investigate this complexity, chemically induced colitis models—particularly the DSS model—have been extensively utilized in inflammatory bowel disease research. In this model, administration of 3% DSS in drinking water for 6–10 days effectively induces acute colitis in rodents (21). *P. notoginseng*, a traditional Chinese herb, has been widely applied in the management of metabolic and inflammatory conditions, including diabetes, osteoporosis, and colitis (22). In the present study, we established a UC model by DSS induction in female ICR mice to evaluate the therapeutic potential of *P. notoginseng* extract. Successful model induction was confirmed by characteristic clinical features in the DSS group, including lethargy, dull fur, bloody diarrhea, and anal bleeding. Treatment with *P. notoginseng* extract significantly ameliorated these physiological and clinical symptoms, indicating its protective role in UC. These findings align with previous reports on other plant-derived therapeutics. For instance, Shaoyao decoction (23), gastrodin (24), and matrine (25) have also demonstrated efficacy in improving UC-related symptoms such as body weight loss and elevated DAI scores. Collectively, our results underscore the therapeutic value of botanically sourced natural compounds in managing UC.

To further elucidate the therapeutic mechanism of *P. notoginseng* extract, we employed LC-MS-based non-targeted metabolomics to profile the fecal metabolome (26). The analysis revealed that *P. notoginseng* extract intervention significantly reversed DSS-induced metabolic disturbances by normalizing the levels of 21 key differential metabolites. Specifically, the extract downregulated several pro-inflammatory and oncogenic metabolites that were elevated in the DSS group. Such as LysoPI (16:0/0:0), PI (16:1(9Z)/18:1(12Z)-2OH (9,10)), N-(8-Amino-1-carboxyoctyl)-alanyl-proline, and N-Eicosapentaenoyl Phenylalanine and MG (6 keto-PGF1alpha/0:0/0:0). Notably, LysoPI (16:0/0:0) (27), which is known to activate the pro-inflammatory GPR55 receptor and is implicated in colonic carcinogenesis (28), was reduced to near-normal levels (29). Interestingly, the study by Bian et al. found that the level of MG (6 keto-PGF1alpha/0:0/0:0) decreased in the plasma of patients with active UC compared to patients in remission with UC and normal controls. Concurrently, *P. notoginseng* extract restored the levels of potentially protective metabolites (30), such as the anti-inflammatory alkaloid Castanospermine (31)and the NF-κB inhibitor Solasodine (32). The coordinated normalization of these metabolites, which are involved in critical processes like immunometabolism and inflammation, demonstrates that *P. notoginseng* extract rectifies the underlying metabolic chaos of UC, thereby contributing to the restoration of colonic homeostasis.

Previous studies have indicated that *P. notoginseng* extract modulates gut microbial diversity and richness (33). Building on this evidence, we conducted a systematic analysis of microbial abundance variations across different taxonomic levels. At the phylum level, the gut microbiota in all experimental groups was primarily dominated by Firmicutes and Bacteroidetes, confirming established ecological patterns. This structural consistency with prior reports by Ling et al. (34) suggests that the therapeutic mechanism of *P. notoginseng* extract operates not through fundamental restructuring of the dominant phyla, but via precise modulation of their relative abundance and functional balance. Notably, *P. notoginseng* extract treatment effectively reversed the DSS-induced elevation in Verrucomicrobiota abundance. Meanwhile, increased Deferribacterota levels were observed in certain individuals, a phenomenon consistent with Luo et al.’s findings (35) that may reflect host-specific variation. Within the Verrucomicrobiota phylum, *Akkermansia muciniphila* has been extensively documented for its therapeutic potential in UC. Previous studies demonstrate that specific *A. muciniphila* strains ameliorate colitis through multiple mechanisms: strain BAA-835 significantly improves acute colitis symptoms (36), other strains enhance retinoic acid synthesis and IL-22 production (37), and specific bacterial components regulate epithelial function through HDAC5-DAB2 signaling pathways (38). Furthermore, *P. notoginseng* extract significantly modulated the abundance of Bacteroidales (order level) and *Ligilactobacillus* (genus level). The observed upregulation of *Ligilactobacillus* aligns with reports showing that *Codonopsis pilosula* polysaccharides similarly alleviate DSS-induced colitis while increasing *Ligilactobacillus* and *Akkermansia* abundance (39). We also noted pronounced increases in *Mammaliicoccus* and *Aerococcus* following *P. notoginseng* treatment. Although the mechanistic basis for these changes requires further investigation, these findings suggest that *P. notoginseng* may selectively promote the growth of specific bacterial taxa, opening new avenues for understanding plant-microbe interactions in UC management.

Our integrated multi-omics approach identified a significant positive correlation between Bifidobacterium abundance and target metabolite concentrations, such as Etamiphylline and 3-Hydroxyisobutyric acid. Etamiphylline and 3-Hydroxyisobutyric acid. As a well-established probiotic genus typically depleted in UC patients, *Bifidobacterium* contributes to intestinal health through multiple protective mechanisms, including enhancement of epithelial barrier function, suppression of pathogenic bacteria, and immunomodulation. The positive correlation between Bifidobacterium and Etamiphylline is particularly intriguing (40). While the precise origin and function of Etamiphylline in the gut remain unclear, it may represent a microbial-derived metabolite or a host metabolite shaped by bacterial activity. Its association with a beneficial bacterium suggests a potential role in mediating some of the probiotic effects, possibly involving immunomodulatory or barrier-strengthening pathways. This observation opens a promising avenue for future research to elucidate the exact source and biological function of Etamiphylline in UC. Specifically, *Bifidobacterium longum* has demonstrated therapeutic potential in inflammatory bowel disease (41), while *Bifidobacterium infantis* acts synergistically with 3’-sialyllactose to ameliorate gut inflammation (42).Similarly, several lactobacilli species—recognized for their anti-inflammatory properties—have shown efficacy in UC models. *Lactobacillus johnsonii* (43) and *Lactobacillus reuteri* (44) represent notable examples of such beneficial strains. These significant microbiota-metabolite correlations, as highlighted in **Figure 11**, suggest that the therapeutic effects of *P. notoginseng* extract are not merely coincidental but may involve a sophisticated crosstalk where the extract modulates key bacterial populations, which in turn influence the production or regulation of specific metabolites, collectively contributing to the alleviation of intestinal inflammation. However, the functional relationships between these differential microorganisms and the associated metabolite alterations identified in our study remain unvalidated, warranting further mechanistic investigation. While *P. notoginseng* extract demonstrates clear potential in restoring gut microbiota homeostasis and modulating colonic gene expression profiles in UC, a more comprehensive understanding of its precise regulatory mechanisms awaits future research.

While *P. notoginseng* extract demonstrates clear potential in restoring gut microbiota homeostasis and modulating host metabolism, its translation into clinical practice requires consideration of its safety profile, bioavailability, and current research status. As a natural product, *P. notoginseng* is generally considered safe at appropriate doses (45), though high doses or long-term use may have potential side effects, such as gastrointestinal discomfort or, rarely, allergic reactions. Its bioavailability can be variable, influenced by factors such as the composition of its complex saponins and interaction with gut microbiota. Some saponins are metabolized by gut bacteria into more absorbable forms, which may partly explain its systemic effects. Clinical studies on *P. notoginseng* for UC are still in early stages, with most evidence derived from preclinical models or traditional use (46). Compared to conventional therapies like aminosalicylates (47) or biologics (48), *P. notoginseng* offers a multi-targeted, holistic approach with potentially fewer side effects, acting not on a single pathway but on the entire “gut microbiota-metabolism-barrier” axis. This makes it a promising complementary or alternative option, especially for patients seeking natural interventions or those with inadequate responses to conventional drugs.

This study provides compelling multi-omics evidence for the therapeutic effects of *P. notoginseng* extract in UC. However, it is essential to acknowledge its limitations. While we revealed significant correlations between gut microbiota, metabolites, and barrier function, the precise causal relationships remain to be established. For instance, whether the observed microbial changes drive the metabolic shifts or vice versa require validation through functional experiments, such as fecal microbiota transplantation (FMT) or targeted metabolite administration in gnotobiotic models. Furthermore, the specific contributions of individual components in the extract to the observed effects need further elucidation. Future research should focus on isolating key active compounds, validating the causality in the microbiota-metabolite-barrier network, and conducting well-designed clinical trials to confirm the efficacy and safety of *P. notoginseng* extract in UC patients.

Additionally, our integrated multi-omics analysis further elucidates the mechanisms by which *P. notoginseng* extract alleviates UC through the remodeling of the gut microbiota. We found that PN intervention not only significantly increased the relative abundance of beneficial bacteria such as Bifidobacterium and Lactobacillus but also reversed DSS-induced dysbiosis, as evidenced by the normalization of the Firmicutes/Bacteroidota (F/B) ratio and the suppression of conditionally pathogenic bacteria like Verrucomicrobiota. This structural remodeling of the microbiota was closely associated with changes in host metabolites, particularly the positive correlation with anti-inflammatory metabolites such as Etamiphylline and 3-Hydroxyisobutyric acid. This suggests that PN may indirectly enhance intestinal barrier function and suppress inflammatory responses by promoting the production of probiotic-derived metabolites. For instance, probiotics like Bifidobacterium and Lactobacillus have been demonstrated to enhance the expression of tight junction proteins (e.g., ZO-1, Occludin), promote mucus layer integrity, and modulate host immune responses, thereby synergistically alleviating the pathological process of UC across multiple dimensions. Therefore, *P. notoginseng* extract may systematically regulate the intestinal microenvironment via the “microbiota-metabolite-barrier” axis, ultimately achieving its therapeutic effects against UC. Future studies employing fecal microbiota transplantation (FMT) or bacterial strain colonization experiments are warranted to further validate the causal role of these specific microbial changes in UC alleviation.

Our findings indicate that *P. notoginseng* extract mitigates DSS-induced ulcerative colitis through a multi-targeted mechanism centered on the coordinated regulation of gut microbiota, host metabolism, and intestinal barrier function. Our findings provide a scientific foundation for developing *P. notoginseng* extract as a natural therapeutic agent for UC, while also illustrating the integrated regulatory capacity of traditional Chinese medicine in addressing complex disease networks. Although further validation is required to fully elucidate its precise molecular mechanisms, this work establishes *P. notoginseng* as a promising candidate for colitis treatment and offers valuable insights for future investigation of its therapeutic applications.

## Data Availability

The datasets presented in this study can be found in online repositories. The names of the repository/repositories and accession number(s) can be found in the article/supplementary material.
